# Retrospective clinical follow-up of implants placed in edentulous jaws after computer-guided surgery and immediate loading, in geriatric patients

**DOI:** 10.4317/medoral.26837

**Published:** 2024-12-24

**Authors:** Loreto Monsalve-Guil, Eugenio Velasco-Ortega, Ivan Ortiz-Garcia, Nuno Matos-Garrido, Jesús Moreno-Muñoz, Enrique Núñez-Márquez, José Luis Rondón-Romero, José López-López, Álvaro Jiménez-Guerra

**Affiliations:** 1 Comprehensive Dentistry for Adults and Gerodontology. Master in Implant Dentistry. Faculty of Dentistry. University of Seville; 2 Department of Odontoestomatology, Faculty of Medicine and Health Sciences (Dentistry), University of Barcelona, L'Hospitalet de Llobregat,Barcelona, Spain; Oral Health and Masticatory System Group, IDIBELL (Bellvitge Biomedical Research Institute), Barcelona, Spain; Facultative Director and Head of Service of the Medical-surgical area of the Dental Hospital of the University of Barcelona

## Abstract

**Background:**

Although there are many works analyzing the clinical behavior of immediate loading of implants inserted by guided surgery, the literature referring specifically to elderly patients is scarce. The aim of this investigation is to present the clinical outcomes of immediate loading of implants inserted by guided surgery in geriatric patients with edentulous maxilla.

**Material and Methods:**

The clinical data of 20 elderly patients with edentulous jaws are analyzed retrospectively. All were diagnosed with cone beam computed tomography, oral examination, and articulator-mounted models to analyze the intermaxillary relationship. They were treated with 4 to 10 implants by flapless guided surgery with immediate loading. After surgery, the implants were loaded with a temporary acrylic prosthesis and six months later, a definitive full-arch ceramic prosthesis was placed.

**Results:**

Twenty patients with an average age of 68.8 years (11 males and 9 females) underwent treatment with a total of 139 implants. Among them, 15 patients (75%) had a history of periodontitis. Ten patients (50%) smoked. Thirteen patients (65%) report a history oof systemic disease. The mean follow-up was 53.1 ± 21.4 months. The clinical outcomes indicated a 100% success rate for the implants. Twenty full-arch fixed maxillary rehabilitations were performed, with an average marginal bone loss of 1.37 mm (S.D. 0.53 mm.) Marginal bone loss was significantly greater for patients with a longer period of clinical follow-up. Peri-implantitis is reported in 17 implants (12.2%) in 8 patients (40%). Four patients (20%) showed some kind of mechanical prosthodontic complications.

**Conclusions:**

The present work indicates that treatment with implant-supported fixed full-arch prostheses, in geriatric patients with edentulous jaws and through guided surgery and immediate loading, this implant protocol seems to be a successful.

** Key words:**Guided implant surgery, immediate loading, edentulous maxilla.

## Introduction

Full arch rehabilitation with several dental implants, digital planning and guided surgery, according to the literature, is a valid dental treatment option (1). This clinical approach contributes to achieving very favorable functional and aesthetic results. Nevertheless, the literature regard-ing elderly is scarce. As reported among others, the study by Lerner *et al* (1), only 3 of the 12 patients analyzed were over 60 years old. Or the work of Lerner *et al*, (2) with only 13 patients and a mean age of 70 years; or that of Makarov *et al*, (3) which presents a sample of 10 patients with a mean age of 63.71 ± 14.55 years (range= 33-77 years). The development of cone beam computed tomography (CBCT) and software that allows the dentist to study the bone and soft tissue architecture in its three dimensions, allows virtual planning of the placement of dental implants (4).

The CBCT provides a noninvasive method to scan maxillofacial bone and 3-D surgical planning software allow to analyze the patient's anatomic information and prosthetic parameters, as well as plan the most appropriate position for the implant.

The use of CBCT-based implant planning can to assess bone volume and quality of alveolar ridges with high precision in the edentulous archs (5). The rapid prototyping can to produce guided templates which, transferring virtual planning to the operative field, are especially useful in edentulous jaws lacking anatomic landmarks for surgical reference (6,7).

Digital technology is increasingly used in the interdisciplinary dental treatment process of edentulous patients, realizing a precise and minimally invasive technique, with predicTable results. Moreover, to obtain successful restorative outcomes is necessary a correct preoperative planning to avoid the invasion of anatomical structures and to insert adequately the implants for a functional and esthetic full-arch restoration (8,9).

Computer-assisted implant surgery aims to virtually reproduce the planned implant positions during surgery. This approach is very important because a correct implant positioning is critical if an esthetically and functionally accepTable restoration that can be maintained through proper oral hygiene is to be achieved (10). Moreover, implant placement must respect the various critical anatomic structures often present in the vicinity of implant sites. Consequently, during diagnosis and treatment planning in guided implant surgery, the surgeon must pay close attention to restorative and anatomic considerations while selecting an alveolar bone site with an adequate quality and quantity to ensure both accuracy and safe implant placement (11).

This less invasive implant technique decreases morbidity because flapless-guided surgery, not needing mucoperiosteal flap elevation and suture, and using all the available residual bone to avoid regenerative procedures (12,13). In fact, reducing the number of surgical procedures, decreasing the intervention and shortening the time between surgery and prosthesis procedures and minimizing the negative impact over the patient (12,13). The overall treatment time and the postsurgical complications (bleeding, hematoma, swelling) are reduced and improving a better patient acceptance and satisfaction than extended to the clinical protocol with good predictability of functional and aesthetic results (11,13). The most important limitation is the lack of visual surgical control as it is a closed surgery (10).

The scientific literature has clearly shown high survival and success rates for implant treatment with computer guided surgery (10,12). According to several studies, edentulous maxillary patients benefit from implant rehabilitation using guided surgery and immediate loading (14,15). Today, immediate loading of implant-supported full-arch rehabilitation has become a routine practice when treating edentulous patients, giving comparable results to conventional and early loading protocols, improving patient acceptance and comfort (16-18). With a clinical computer-assisted protocol of implant placement and immediate loading, it is possible to perform a successful full-arch rehabilitation with functional and aesthetic results (19-21).

Our objective was to analyze the clinical results of fixed full-arch rehabilitations by guided surgery of implants and immediate loading for treating edentulous maxillary patients.

## Material and Methods

This retrospective study involved maxillary edentulous patients who attended for treatment at the Master's in Implantology clinic of the Faculty of Dentistry, University of Seville, Spain, from September 2015 to March 2021. The study was carried out in accordance with the principles described in the Declaration of Helsinki on clinical research in humans. The study followed the STROBE guidelines (18 of 22 items) and was approved by the ethical committee of the University of Seville approved the study (July 11, 2013) and all patients signed the written informed consent for implant placement.

The study population comprised 20 patients (treated consecutively), 11 men and 9 women, aged between 49 and 83 years (mean age 68.8 ± 9.3). Inclusion criteria required that patients be prescribed a complete maxillary implant-supported rehabilitation. Patients were excluded if the clinical history included uncontrolled chronic diseases, particularly diabetes or coagulation disorders, smoking 10 or more cigarettes per day, alcohol and/or drug abuse, bruxism, or uncontrolled periodontal disease. Treatment planning should include taking a detailed clinical history, performing an oral examination with orthopantomography using CBCT, making diagnostic models for intermaxillary relationships, and taking clinical photographs. Patients should have been informed about possible implant treatment options and consented to immediate placement of implant-supported full-arch prostheses using guided surgery.

- Surgery protocol

Following the group protocol (12,21), if the protocol was not met based on the medical history, the case was not included in the data analysis. Before the surgery, patients were given preventive antibiotic therapy (500 mg of amoxicillin and 125 mg of clavulanic acid one hour before implants insertion); they continued with the antibiotic regime postoperatively, taking 3 daily capsules for 5 days. Post-surgery, they were instructed to use a chlorhexidine mouthwash twice a day for 15 days. Patients were prescribed ibuprofen (600 mg, three times a day) for 5 days. All procedures were performed under local anesthesia with articaine and adrenaline [1:50,000].

Each participant underwent cone beam computed tomography (CBCT) (Picasso Master 3D®, Vatech, Gyeonggi-do, South Korea) with a prosthesis immediately after the scan and an occlusal index in place. Implant positions were planned using 3D software (Galimplant 3D®, Galimplant®, Sarria, Spain, Version for Windows and MacOS) to determine the optimal placement considering both prosthetic and alveolar aspects (12,21).

**Figure 1:**
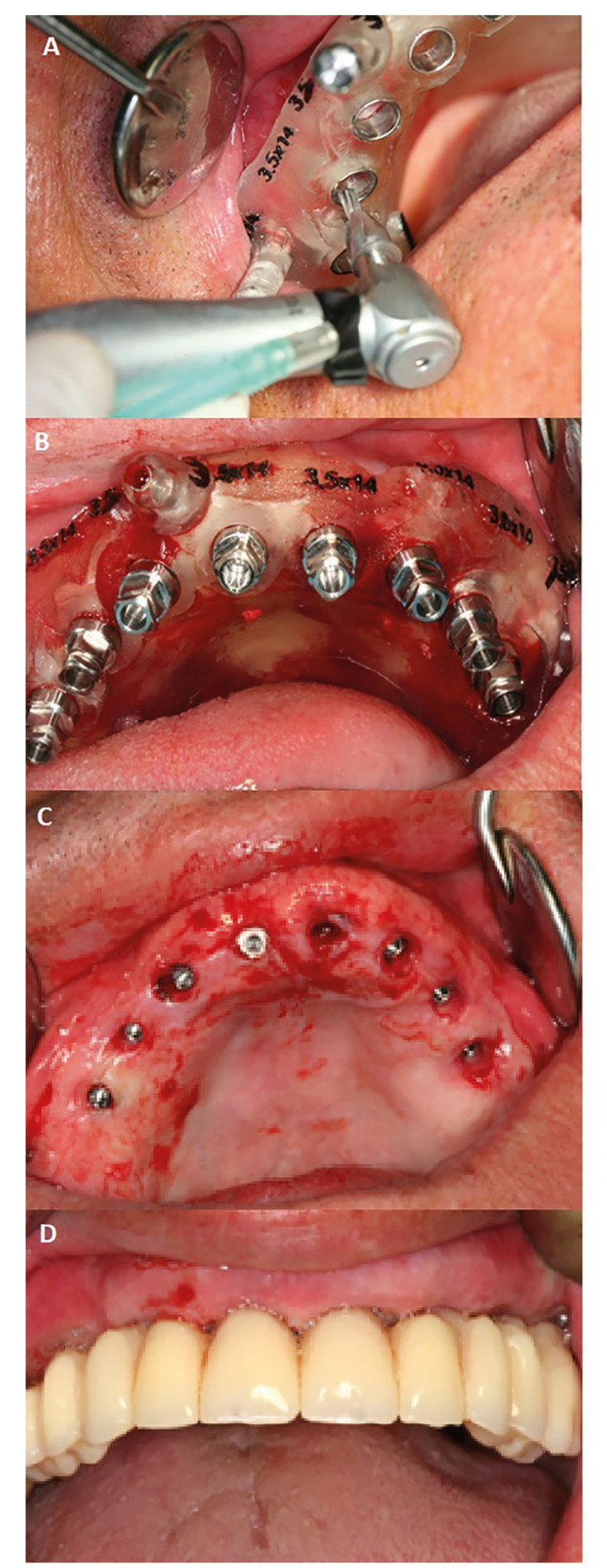
Case 1. 78-year-old patient, smoker and with a history of periodontitis. A) Guided surgery plate and implant insertion B) All implants inserted in one of the cases. C) Surgical aspect immediately after implant insertion. D) Provisional prosthesis inserted in the mouth.

Flapless surgery was executed using a guided template created through digital planning. On the day of procedure, following anesthesia, the surgical template was positioned in the mouth and fixed to the bone using 2 screws in the buccal plates (Fig. 1). The guided surgery involved sequential preparation of implant sited with incrementally larger drills, concluding with the placement of all implants through the guide. Implants were placed starting with the distal ones and moving anteriorly. The implants used were Surgimplant® screw implants (Galimplant®, Sarria, Spain) featuring a hexagonal external connection and a sandblasted, acid-etched surface.

- Post-surgical records 

To assess implant stability after placement, both insertion torque and resonance frequency analyzes were utilized. The insertion torque applied was recorded before the surgical guide was removed. All implants were put in place using the implant motor, an insertion torque of ≥35 Ncm at the time of placement was deemed accepTable [8,14]. Additionally, resonance frequency analysis was conducted to check the stability of each implant immediately after the surgical guide was removed (Penguin RFA, Klockner, Barcelona, ​​Spain). A stability conscious between 55 and 85 was scored as correct (12,21).

- Following and surgical procedure

**Figure 2:**
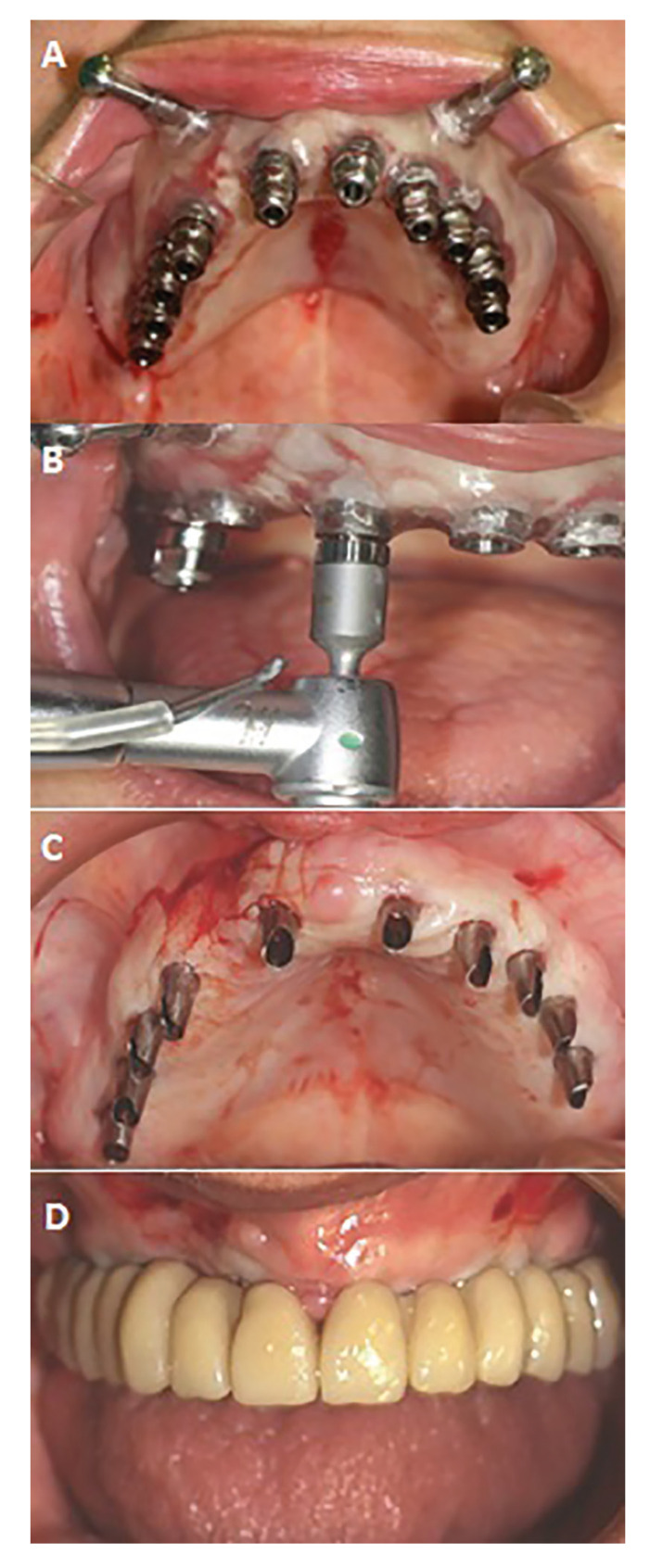
Case 2. 71 years-old and no smoker patient. A) Guided surgery plate inserted with the posterior implants placed. B) Checking the torque of one of the implants. C) Straight abutments for immediate rotational loading prior to insertion of the provisional insertion. D) Provisional prosthesis inserted in the mouth.

Following the surgical procedure, all patients were immediately fitted with transepithelial abutments and a temporary full-arch rehabilitation, straight abutments for immediate rotational loading, Galimplant®, Sarria, Spain) (Fig. 2).

**Figure 3:**
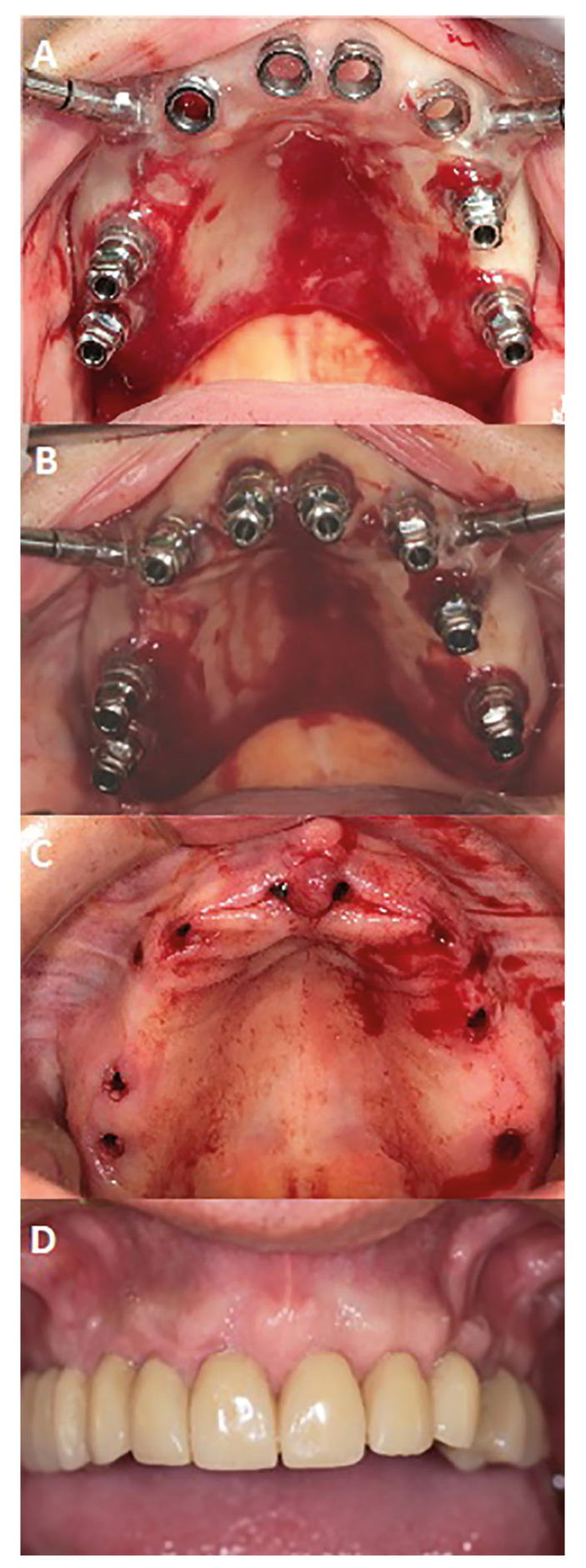
Case 3. 69-year-old patient, smoker with the definitive prosthesis inserted, follow-up of 3 years and 2 months. A) Guided surgery plate inserted with the posterior implants placed. B) Eight implants with their carriers inserted. C) Post-surgical aspect prior to insertion of the provisional prosthesis. D) Definitive prosthesis inserted in the mouth.

Immediate loading was carried out only when an insertion torque of ≥35 Ncm and an ISQ value of ≥55 were achieved. Acrylic-resin cement was used for the temporary restorations of the maxillary arch (Vertys Templus monomasa, Vertysystem®, Vicenza, Italy). Six months post-implant placement, after clinical review and orthopantomography, the temporary restorations were extracted. Impressions were then made using addition silicone material with open individual trays, and definitive metaloceramic full-arch restorations were fabricated and placed onto the dental implants (10,20) (Fig. 3).

The survival of implants was assessed based on their stability and the absence of radiolucency around them, as well as the absence of mucosal suppuration and/or pain. Clinical control is carried out at 3 and 6 months post-implant placement, and annually from 1 to 8 years after guided surgery. During these follow-ups, patients underwent dental hygiene assessments, clinical and radiological reviews of the prostheses and implants. Marginal bone loss was monitored using digital periapical radiographs which were taken perpendicular to the long axis of the implants. Differences between the 1-year follow-up radiograph and the baseline radiograph were compared.

The preparation of the definitive prosthesis is carried out based on the passivity test through radiographs. In case of discrepancies between the model and the clinical situation, the necessary adjustment is made prior to manufacturing the metal-ceramic structure. On the other hand, in our group, an annual follow-up of the prosthesis is established, with its disassembly if necessary. Likewise, all patients are instructed in hygienic control of the prosthesis with complementary oral hygiene methods (preferably irrigators and interproximal brushes). In each follow-up visit, reinforcement of home maintenance is carried out.

- Statistical analysis

All data obtained were analyzed using the SPSS software package (SPSS 11.5.0, SPSS, Chicago, USA). Descriptive statistics were used to present the results. Data related to qualitative variables were expressed in absolute terms as well as percentages, and were calculated using the chi-square test. For the analysis of quantitative variables, means, standard deviations (SD), medians, ranges and 95% confidence intervals (CI) were used. Group similarities were confirmed using analysis of variance (ANOVA). ANOVA will be used to compare between groups when the analyzed variables meet the following requirements: i.-The analyzed variable has a normal. ii.-The K samples on which the treatments are applied are independent. iii.-The populations all have equal variance (homoscedasticity). The non-parametric Mann-Whitney U test was used to compare differences between groups based on various measured risk factors. This test was used when the data is not organized in a normal way. Statistical significance was set at *p* < 0.05.

## Results

Aspects related to the results are shown in the Tables 1, 2 and 3. 139 implants were placed in 20 fully edentulous maxillary patients, comprising 11 males and 9 females. Statistical analysis revealed no significant differences related to sex and age (chi-square test, *p*=0.65310). Only two patients, aged 49 and 60, were under 65 years old. Among the patients, 15 (75%) had a history of periodontitis, including 9 males and 6 females (*p*=0.4362). Additionally, 10 patients (50%) were smokers, and 80% of the patients with a history of periodontitis were also smokers (*n*=8) (Table 1). Nine smoking patients (90%) were under 70 years old; These differences were statistically significant (chi-square test, *p*=0.00035). Thirteen patients (65%) suffered from a systemic disease (i.e. hypertension, heart failure, diabetes, osteoporosis). Ten patients with systemic diseases (76,9%) are over 70 years old; These differences were statistically significant (χ² test, *p*=0.00103).

Of the 139 implants placed in the maxilla, 3 patients (15%) patients received 10 implants, 7 patients (35%) received 8 implants, 3 patients (15%) received 7 implants, 2 patients (10%) received 6 implants, and 5 patients (25%) received 4 implants. The average follow-up period was 53.1 ± 21.4 months (ranged: 36-103 months). All implants (100%) had a diameter of 4 mm. Sixty-six implants (47.5%) were 12 mm in length, 56 (40.3%) were 10 mm and 17 (12.2%) were 8 mm. No implants were lost during the treatment. The mean insertion torque was 42 Ncm (range 35 to 60) and the mean ISQ value was 60 (range 55-70).

The cumulative survival rate for all implants was 100%. (Table 2).

During the follow-up period, 19 implants (13.7%) out of the 139 implants in 9 patients (45%) were associated with mucositis. In terms of peri-implantitis, 17 implants (12.2%) out of the 139 implants in 8 patients (40%) were affected. In patients with a history of periodontitis (75%), peri-implantitis was more prevalent, and it was also among smoking patients (50%). It was especially significant if they were smokers and had periodontal disease (40%). The relationship between peri-implatitis and a history of periodontitis, smoking, or both aspects together, is presented in Table 3, with peri-implantitis being significant with the combined history of smoking and history of periodontitis.

The average marginal bone loss was 1.37 mm (± 0.53 mm standard deviation), with a range from 0 to 2.5 mm during the follow-up evaluation. Marginal bone loss was 1.91 ± 0.37 mm for patients with a longer follow-up period (≤45 months) and 1.14 ± 0.41 mm for patients with a shorter follow-up period (≥46 months), showing statistical significance (ANOVA; *p*=0.0010). If we analyze the bone loss of the first 4 years of follow-up, the data are not significant, with a *p*=0.562 (Kruskall-Wallys test); thus, at one year of follow-up we obtain 1mm (SD=0.538); at two years 1.2mm (SD=0.274); 3 years 1.33mm (SD=0.289) and 4 years 1.37mm (SD=0.53). Marginal bone loss and its relationship with different patient conditions are presented in Table 4.

In terms of prosthetic outcomes, a total of 20 maxillary fixed full-arch rehabilitations were placed. Four patients (20%) experienced some form of technical prosthodontic complications with the definitive prosthesis, such as ceramic chipping and screw loosening. Two of them at one year of follow-up and the other two at the third year of follow-up. Of the 20 rehabilitations, a total of 6 (30%) were dismantled for maintenance due to poor hygiene. One of them on three occasions, one year, two years and the third year. Two in the first and second year and three only once.

The clinical follow-up (grade of patient hygiene), the need for more adjustments during the making of the definitive prosthesis and/or the history of associated medical illnesses do not represent differences in terms of the studied parameters.

## Discussion

This study analyzed the clinical outcomes of planning and treating geriatric edentulous maxillary patients using guided surgery for full-arch implant rehabilitation with immediate loading (22,23). Although this technique, fixed rehabilitation on implants associated with guided surgery, has improved the planning of complex cases, in elderly patients we have little evidence (1-3).

Computer-guided implant protocols can help implantologist perform successful implant therapy while minimizing or avoiding flap elevation. Additionally, three-dimensional diagnosis using CBCT allows dentist to reduce the risk of damaging nearby structures, particularly in maxillary areas who have limited residual bone. In these patients, CBCT planning can be employed for edentulous maxillary patients with anatomical limitations, such as the maxillary sinus (23,24). Moreover, in specific cases, this approach can eliminate the need for a bone grafting procedure.

The clinical results of this study demonstrate that guided surgery for treating edentulous maxillary patients is a highly favorable option for dental implants. Notably, 100% of the implants survived during the follow-up period. Scientific evidence suggests that implant insertion using guided surgery is at least as successful as conventional implant surgery (17-19,25). Some studies have reported that implant placement with guided surgery is more accurate than freehand surgery (11). A recent randomized controlled trial comparing the accuracy of immediate implant placement with guided surgery versus freehand found that the freehand group (control) had greater crestal, apical, and angular deviations compared to the guided surgery group (test) (11). In a clinical study conducted on twenty-six patients, they were randomly assigned to conventional or guided surgery. In the test group, the implants were placed in the upper jaw, using a surgical guide with a minimally invasive flap, and loaded immediately. In the control group, the implants were inserted by open flap surgery with a prosthetic stent and loaded immediately (18). A total of 70 implants were placed (34 in the control group and 36 in the test group). One implant failed in the guided surgery group. The cumulative failure rate was 1/36 (2.7%) for the guided surgery group and 0/34 (0%) for the conventional surgery group. Thus, this study demonstrated that implants can be successfully integrated into the posterior maxilla, using a guided surgical approach with immediate loading (18).

Several clinical studies have indicated that digital planning and guided surgery for complete-arch rehabilitations on multiple dental implants are a valid treatment approach (25,26). A retrospective study reported on the effectiveness of complete-arch rehabilitations with screw-retained fixed prostheses supported by four dental implants inserted using a fully guided surgical protocol. In total, 160 implants were placed in 37 patients, with three patients receiving rehabilitations in both arches. Patients were followed up to 4 years to evaluate overall treatment success. In total, 40 full arch rehabilitations were performed, 26 in the maxilla and 14 in the mandible. Only five implants failed, with an overall implant survival rate of 96.9%. No definitive prosthesis failed, with a 100% success rate (26).

Another retrospective study evaluates the clinical and radiographic results of follow-up up to ten years, of immediate full-arch prostheses, supported by 4/6 implants, performed with flapless surgery and planning based on immediate loading and guided surgery (13). Twenty-eight edentulous patients were treated with 32 prostheses (17 all-on-4/15 all-on-6) and 164 implants. Of 33 prostheses, 17 were all about four (eight in the mandible/nine in the maxilla) and 16 were all about six (six in the mandible and 10 in the maxilla). The cumulative implant survival rate was 89.7% for the All-on-four system (there were seven failures) and 99.0% for the All-on-six (one failure). The prosthesis survival rate was 88.2% for the All-on-four system. No failures were recorded in the All-on-Six (13). Guided implant surgery enhances precision in implant placement, especially in fully edentulous cases, leading to significantly reduced surgery duration, improved postoperative clinical conditions, and the option for immediate loading with provisional restorations (14-21). According to findings from various studies, rehabilitating the edentulous maxilla with an immediately loaded implant-supported prosthesis following a guided surgery protocol has shown long-term success (10,14,15,18). In the present study, 20 maxillary edentulous patients received a total of 139 implants inserted via flapless guided surgery and immediately loaded with provisional fixed full restorations. Following a 6-month provisionalization period, 20 definitive fixed full-arch restorations were delivered, with no implant failures observed during the mean clinical follow-up period of over 4 years.

In our present study, follow-up visits were scheduled after implant and prosthetic placement. All patients were recalled for evaluation of peri-implant soft tissue conditions, individual implant stability and radiographic marginal bone loss. However, marginal bone loss increased significantly as the clinical follow-up period increased from 1.14 ± 0.41 mm in patients with the shortest follow-up time to 1.91 ± 0.37 mm in those with the longest clinical follow-up period. A similar trend is reported by a retrospective study on clinical and radiographic outcomes up to 10 years of follow-up with immediate rehabilitation of edentulous jaws by guided implant surgery. Mean value of marginal bone loss was 1.38 ± 0.1.28 mm at 5-year and 2.09 ± 0.56 mm at 10-year follow-up (13).

Assessing the soft tissue conditions and marginal bone changes in immediately loaded implants, placed in edentulous jaws using guided surgery, is a crucial factor for the long-term success of this treatment (13,21,27-28). A clinical study in 21 edentulous patients showed a mean probing depth of 2.8 mm in the maxilla at 19 months of mean follow-up (28). Although it indicated that a probing depth of 3 mm can be found around successful implants, the diagnostic value of probing around implants remains uncertain. In the maxilla, marginal bone loss was measured at -1.17 mm (SD = 1.23) (27). More recently, a study reported the results of 13 fully edentulous maxillary patients treated for virtual treatment planning as well as flapless implant placement. The mean follow-up time was 9.1 years. The results showed a mean bone loss was 0.91 (28).

The occurrence of complications is a critical aspect in the long-term care of patients treated with implants via guided surgery (29). A recent study involving 368 implants in 229 patients reported an overall implant survival rate of 97.04% at the 5-year follow-up. However, early implant failure was recorded at 1.07%. Peri-implant mucositis was observed in 48.2% of the implants, with 15 cases of peri-implantitis (5.5%) detected. Additionally, 48 technical complications, such as abutment loosening, were documented during the follow-up examination after prosthesis loading. These complication rates were consistent with those reported in current literature (30).

Prosthodontic complications were common in the present study, with 4 patients (20%) experiencing technical issues with their restorations, such as ceramic chipping and screw loosening. However, these minor complications did not affect the survival of the prostheses. In fact, technical complications are relatively common in studies involving edentulous patients who undergo guided surgery and immediate loading for full-arch rehabilitations (12-14,21,28,29). The incidence of technical complications can be related with several factors as treatment planning, number of implants, type of prostheses material (i.e. resin, ceramic), bruxism, and maintenance program with periodical recalls. Several complications as chippings, detachments and wear of prosthetic structures are frequent, but these technical implications are easily solved in the laboratory or in the clinic with repair or replacement of prosthetic abutments (12-14,21,28,29). Technical complications are more frequent in edentulous patients treated with All-on-four or All-on-six approach because there is a higher overloading over the prosthetic structure with less implants and the presence of cantilevers in the posterior area of maxilla (13,23,28).

In the present study, the incidence of peri-implant diseases was an important biological complication. One important finding during the clinical examination of the soft tissues in the present study was the presence of peri-implant mucositis, reported in 19 implants (13.7%) of the 139 implants in 9 patients (45%). In such cases, limited accessibility for oral hygiene instrumentation at implant sites can lead to increased plaque accumulation and subsequent peri-implant inflammation. In fact, several studies of rehabilitations of edentulous patients by guided surgery, have shown amounts of plaque accumulation and calculus on the basal surface of the prosthetic suprastructure (23). Moreover, the cemented full-arch rehabilitation can increase the risk of cement-related mucositis and peri-implantitis (27).

During the follow-up period, 17 implants (12.2%) in 8 patients (40%) developed peri-implantitis (Table 3). Peri-implantitis was more common in patients with a history of periodontitis (75%) and among smokers (62.5%). These findings align with several studies on implants placed in fully edentulous patients, where flapless-guided surgery and immediate loading with fixed prosthetic rehabilitations were utilized (20,30). Implant treatment in patients with a history of periodontitis has been suggested to have different outcomes compared to patients without such history, with a higher prevalence of peri-implantitis observed in implants placed in fully edentulous patients who have a history of periodontitis (17) Smoking has been linked to a higher rate of peri-implantitis in patients treated with guided implant therapy (27,28). The maintenance of the patients with the prosthesis did not present any difference in the results, probably due to the strict control of this aspect and the reinforcement that the maintenance is done continuously in each of the visits (12).

The most important limitations of this study are that, as it is a retrospective study, we lack a control group and, as a proposal, we believe that it is important to carry out a longer-term follow-up of the patients to assess whether the clinical results are maintained.

In conclusions. Guided implant surgery, with a CBCT diagnosis and the use of surgical guides for the preparation of the implant site, has gained popularity in implant dentistry. This study shows that using guided surgery for full-arch fixed rehabilitations in maxillary edentulous geriatric patients and immediate loading of implants placed appears to be a successful implant protocol.

## Figures and Tables

**Table 1:** Description of patient’s characteristics.

Variables	n	%
Males	11	55
Females	9	45
History of periodontitis	15	75
Smokers	10	50
History of periodontitis and smokers	8	40
Systemic disease	13	65
Full prosthesis wearers	4	20
Totally edentulous for more than 6 months	5	25
Patients who have their remaining teeth removed at the time of implant surgery	14	70

n = patient.

**Table 2:** Description of implant’s characteristics.

Variables		n	%
4 mm implant diameter		139	100
12 mm implant length		66	47.5
10 mm implant length		56	40.3
8 mm implant length		17	12.2
Number of implants per patient	10 implants	30 (3 p)	15 (p)
	8 implants	56 (7 p)	35 (p)
	7 implants	21 (3 p)	15 (p)
	6 implants	12 (2 p)	10 (p)
	4 implants	20 (5 p)	25 (p)
Implants inserted in the initially planned position		134	96,4
Patients in whom planning was fully consistent with surgery		18 (p)	90

n = implant. p = patients.

**Table 3:** Description of patients with complications.

Condition	Variables	Positive condition	Negative condition	n	*p*
History of periodontitis	Peri-implantitis	6	2	8	*p=*1
	No peri-implantitis	9	3	12	
	n	15 (75%)	5 (25%	20	
Smoking	Peri-implantitis	5	3	5	*p=*0,36
	No peri-implantitis	5	7	12	
	n	10 (50%)	10 (50%)	-	
History of periodontitis and Smoking	Peri-implantitis	6	2	8	*p=*0,02
	No peri-implantitis	2	9	12	
	n	8 (40%)	12 (60%)	20	
History of periodontitis	Mucositis	5	4	9	*p=*0,65
	No- Mucositis	5	6	11	
	n	15 (75%)	5 (25%	20	
Smoking	Mucositis	5	4	9	*p=*0,65
	No- Mucositis	5	6	11	
	n	10 (75%)	10 (25%	-	
Systemic disease	Mucositis	6	3	9	*p=*0,54
	No- Mucositis	7	6	11	
	n	13	7	20	

* *p* < 0.05. n = patient.

**Table 4:** Marginal bone loss and its relationship with different patient conditions.

Condition	Variable	Positive condition	Negative condition	n	*p*
Smoking	n	10	10	20	*p=*0,84
	Median maginal bone loss	1,35	1,40		
History of periodontitis	n	15	5)	20	*p=*0,72
	Median maginal bone loss	1,4	1,3		
Systemic disease	n	13	7	20	*p=*0,45
	Median maginal bone loss	1,35	1,5		

* *p* < 0.05. n = patient.
